# A giant hand lipoma as a rare cause of secondary carpal tunnel syndrome - A case report

**DOI:** 10.1016/j.ijscr.2020.11.083

**Published:** 2020-11-19

**Authors:** Mariana Barreira, Nuno Marques, Vicente Campos, Guilherme Marques, Sérgio Gonçalves, Sandra Stefanova Alves

**Affiliations:** Centro Hospitalar Universitário de Lisboa Central, EPE, Lisboa, Portugal

**Keywords:** Hand surgery, Carpal tunnel syndrome, Lipoma, Compression neuropathies, Case report

## Abstract

**Introduction:**

Lipomas are a rare cause of compressive neuropathy and they lead to atypical clinical presentation that can mimic carpal tunnel syndrome.

**Case Presentation:**

The authors describe a rare presentation of a carpal tunnel syndrome recurrence after a hand giant lipoma, presenting with rapidly compression neuropathy of the median nerve, 6 months after de primary surgery.

**Discussion:**

Lipomas are common benign soft tumours. Their occurrence in the hand remains rare and they rarely cause secondary entrapment neuropathies. Carpal tunnel syndrome is mostly idiopathic and bilateral. Local factors should be suspected when these neuropathies present with atypical symptomatology or even when they recur after primary conventional surgical release. Investigation should consider images studies as this correct preoperative assessment leads to successful diagnosis and treatment.

## Highlights

• Space-occupying lesions like lipoma are a rare cause of secondary compression neuropathies of the upper extremity.
• Atypical symptomatology should raise suspicion of local causes and regular work-up should be extended.
• Images studies leads to successful diagnosis and guidance of the best surgical treatment.
• Monobloc resection is still the best treatment to reduce the risk of iatrogenic lesions and disease recurrence.

## 1 Introduction

Lipomas are the most common benign soft-tissue neoplasms, accounting for almost 50% of all soft-tissue tumours [1]. They are frequently found on the limbs, but their occurrence in the hand and wrist remains rare, approximately 1–3,8% of benign tumours of the hand [2,3].

Carpal tunnel syndrome (CTS) is one of the most common compression neuropathy [4] and in approximately 50% of the patients the pathogenesis is unclear (idiopathic) [5]. When secondary local underlying causes are present, simple section of the transverse carpal ligament may not be sufficient, and further surgical exploration might be warranted [6]. Space-occupying lesions like lipomas rarely cause secondary compression neuropathies of the upper extremity [7], and may lead to misdiagnosis and treatment errors.

This case report describes a rare presentation of a hand giant lipoma causing secondary carpal tunnel syndrome treated in a tertiary referral public hospital. This work has been reported in line with the SCARE criteria [8].

## 2 Presentation of case

A leucodermic sixty-three-year-old female patient, was referred to an orthopaedic consul presenting with a numbness sensation of the right hand. She had a medical history of hypothyroidism, hypertension and hypercholesterolemia and her medication included levothyroxine, bisoprolol and atorvastatin. The patient was diagnosed and treated for an idiopathic CTS 6 month before, with a palmar mini-open surgical decompression of the median nerve. The patient confirmed complete clinical improvement in the early post-operative period.

**Fig. 1:**
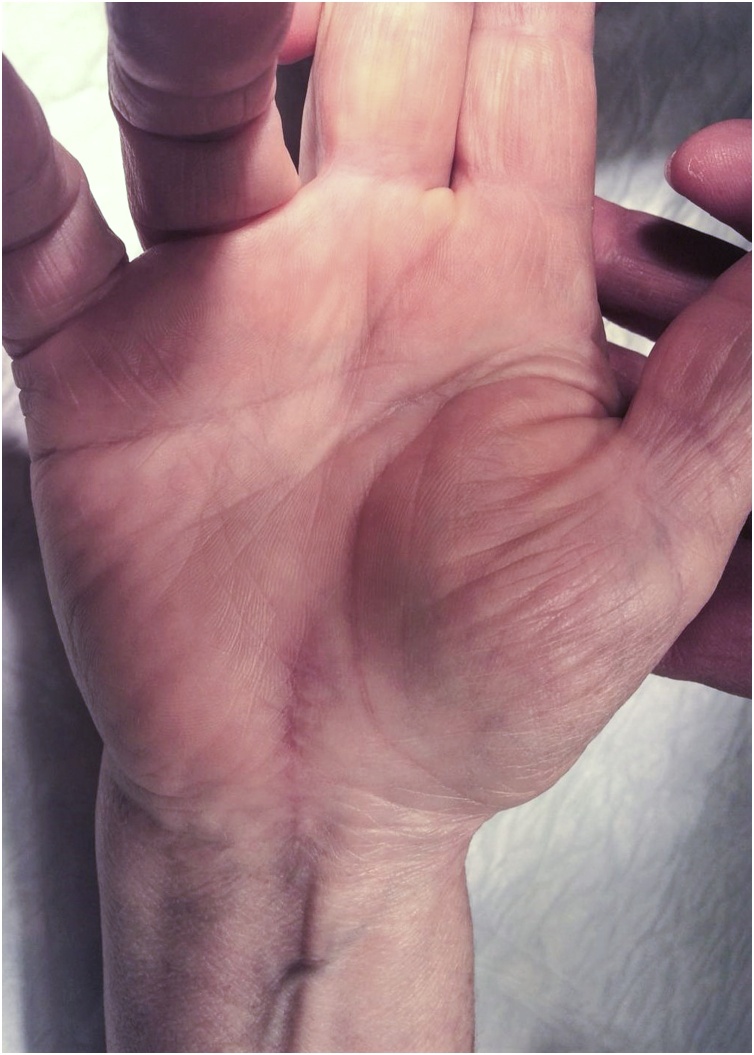
Physical examination of the right hand showing a thenar mass and scar from previous surgical procedure.

On presentation the patient complaint of recurrence of numbness in the median nerve distribution of the right hand. On examination, there was a mass on the thenar eminence (Fig. 1). It was soft on palpation, non-tender, with well-defined edges. The patient experienced pain on flexion and abduction of the thumb, but the neurological examination revealed no differences in motor function and sensation from the contralateral hand.

**Fig. 2:**
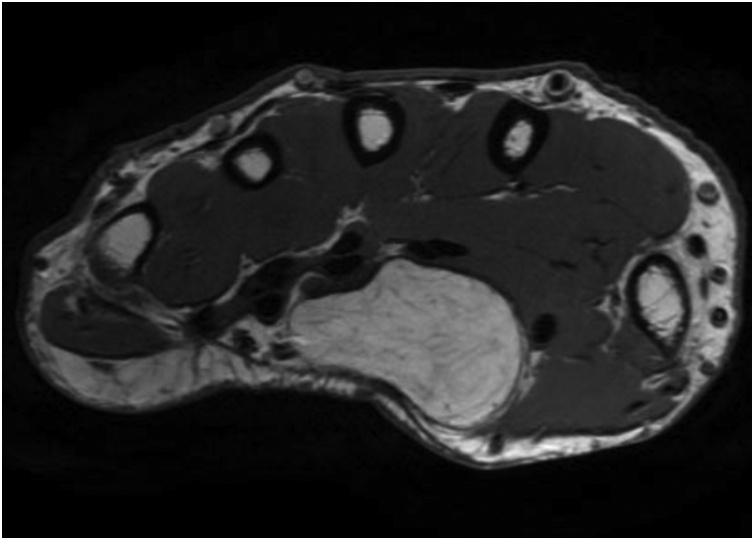
T1-weigthed axial MRI image showing an occupying-space lesion with hypersignal.

**Fig. 3:**
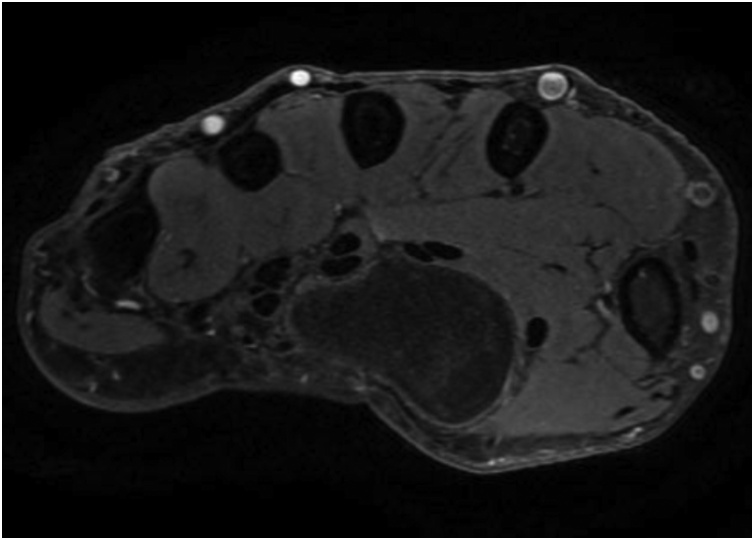
Axial MRI image with fat suppression showing an occupying-space lesion compatible to adipose tissue.

**Fig. 4:**
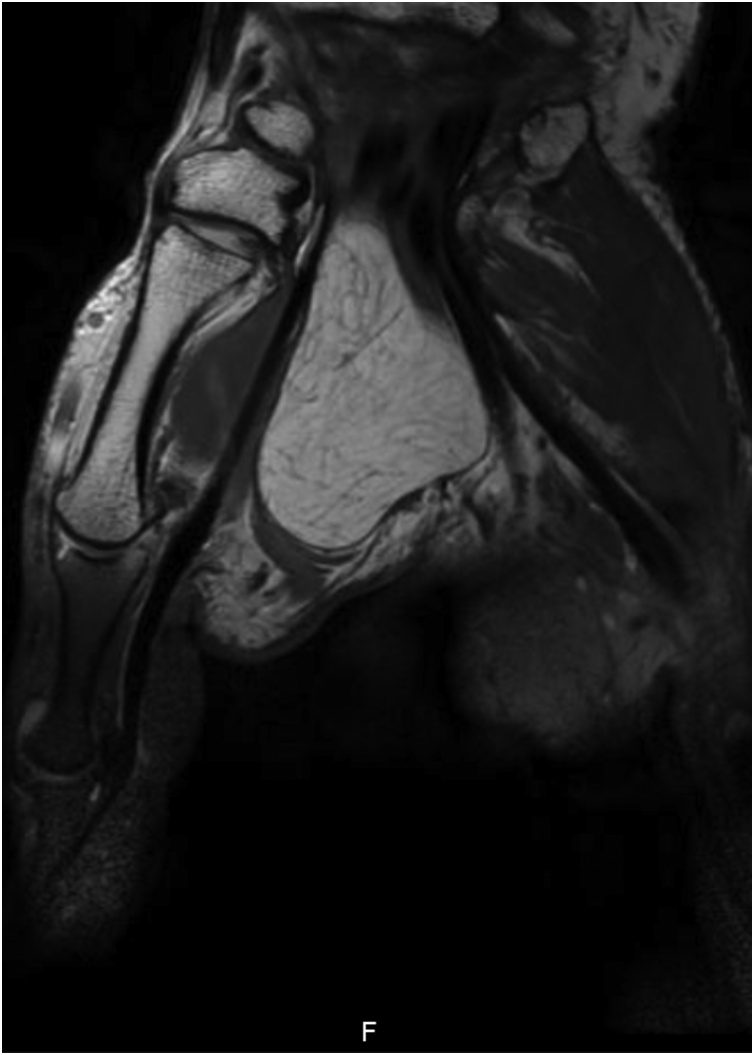
T1-weigthed coronal MRI image showing a capsulated adipose tumour without tendon involvement.

**Fig. 5:**
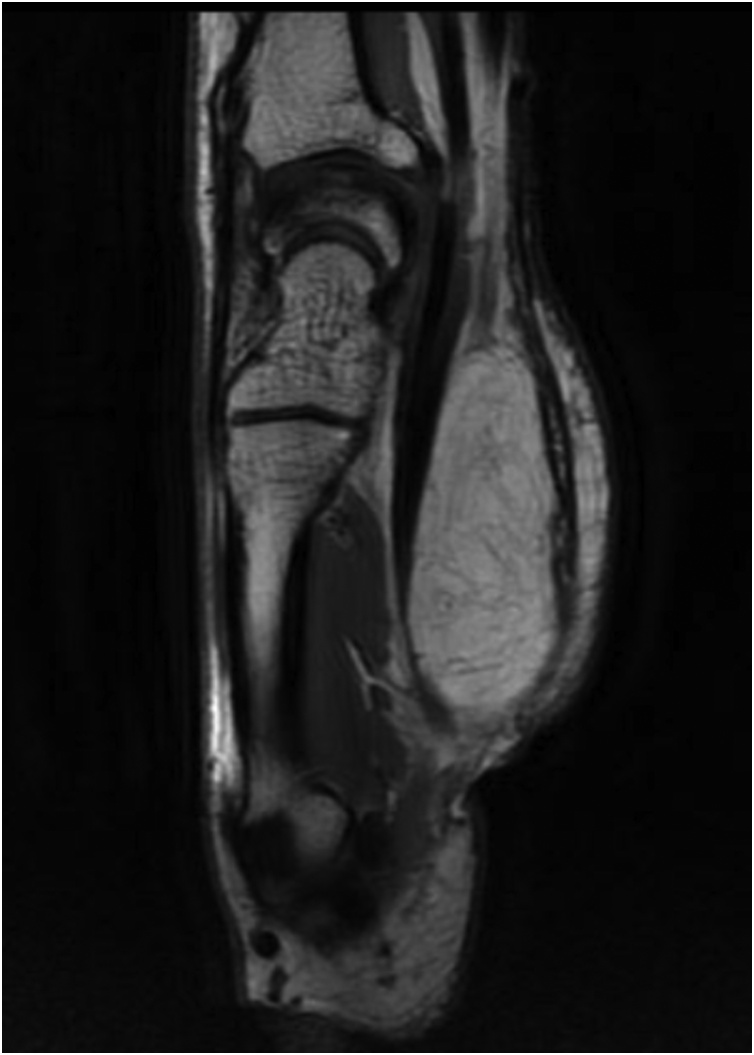
T1-weigthed sagittal MRI image showing a capsulated adipose tumour shaping the flexor tendons, with signs of invasion.

Standard X-rays showed no abnormalities. Ultrasound (US) and magnetic resonance image (MRI) of the hand revealed an occupying-space lesion of adipose density, capsulated with well-defined contours (Fig. 2, Fig. 3). MRI showed no signs of local aggressivity and presented a large homogenous tumour measuring approximately 46 × 31 × 17 mm, shaping the flexor tendons and median nerve, but laying independently from them (Fig. 4). The median nerve presented no focal lesions, but it was distally molded and rectified by the presence of the tumour (Fig. 5). All findings suggested the diagnosis of a lipoma.

**Fig. 6:**
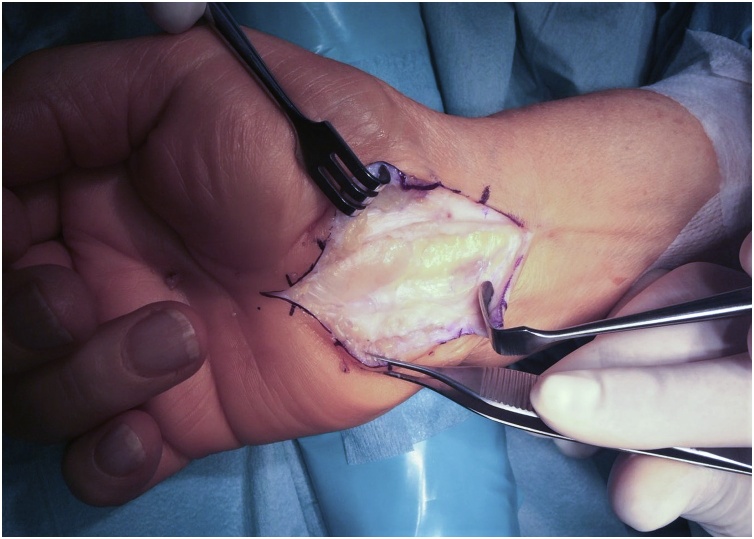
Intraoperative picture. Surgical standard palmar approach, with an intracanal lipoma.

**Fig. 7:**
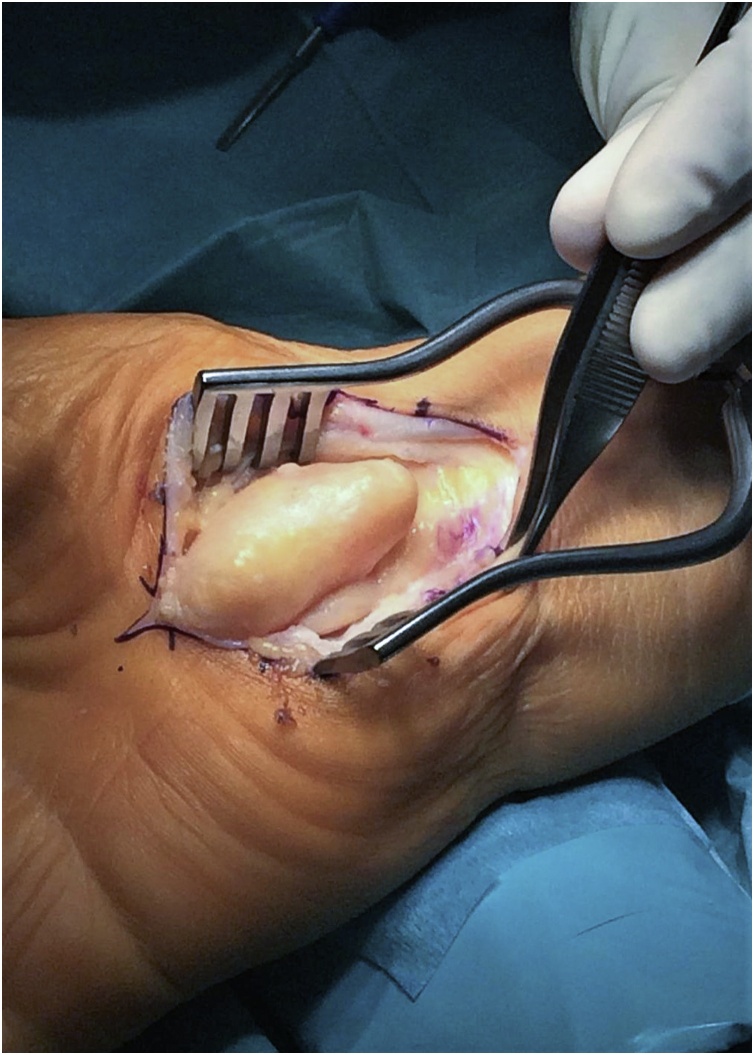
Intraoperative picture. Dissection of intracanal lipoma, laying above the median nerve.

**Fig. 8:**
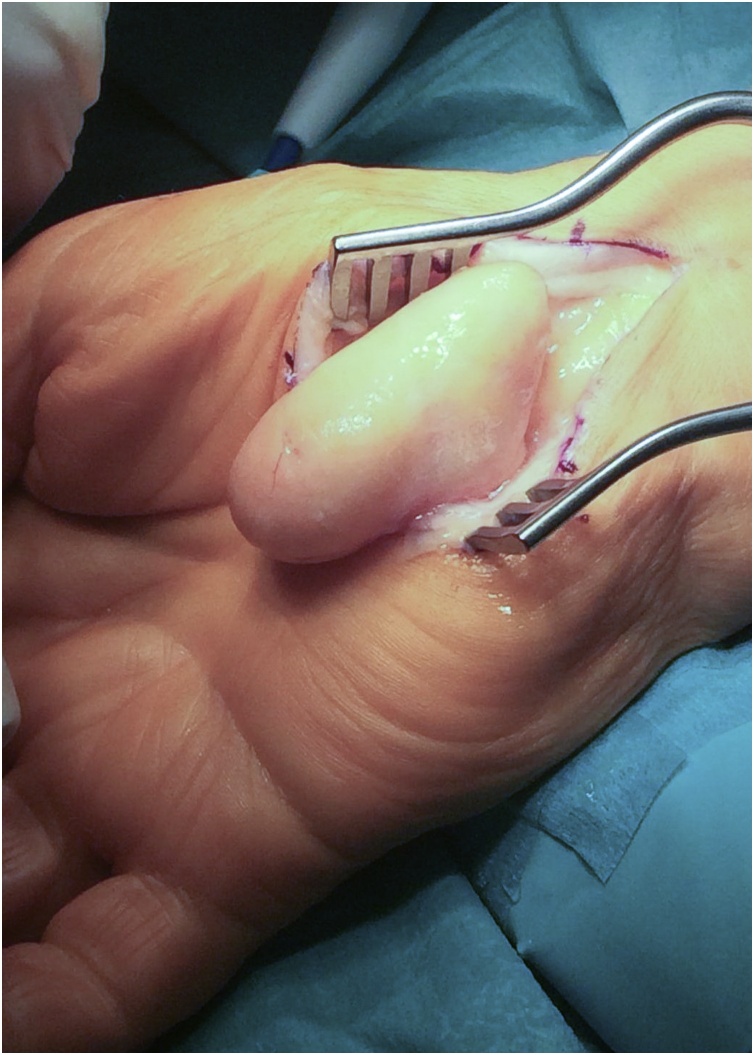
Intraoperative picture. Completed surgical dissection of intracanal lipoma.

**Fig. 9:**
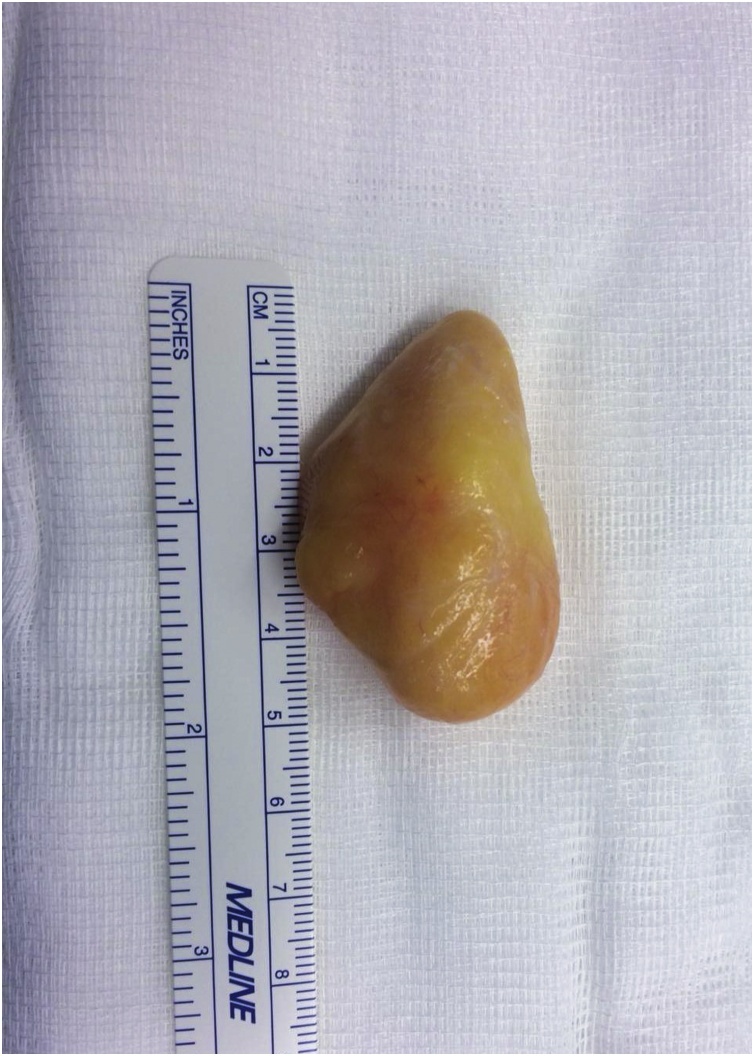
Excised lipoma before fixation.

**Fig. 10:**
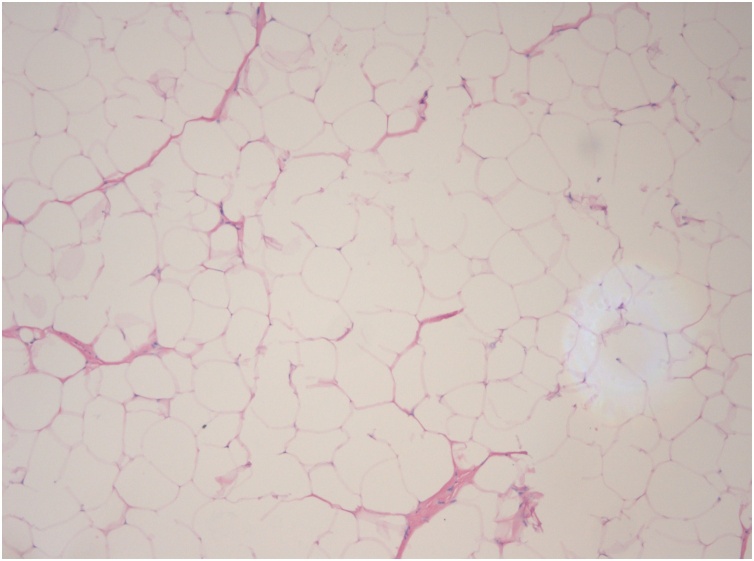
Histopathological analysis showing mature adipose tissue.

Surgical treatment was proposed and executed by a hand specialist, and an excisional biopsy was performed under general anaesthesia. Through a standard palmar approach and anterior annular carpal ligament release, an intracanal giant lipoma was found (Fig. 6). Tumour dissection was complete and an *en bloc* marginal resection carried out (Fig. 7, Fig. 8). The tumour was sent to anatomopathological study using formaldehyde and the skin was closed with non-absorbable sutures. The surgical procedure and early post-operative period underwent with no complications. The histopathological analysis documented a nodular, elastic fragment of soft tissue, loculated and yellow in colour, weighting 13 g and measuring 51 × 35 × 25 mm (Fig. 9). The lesion was constituted by mature adipose tissue (Fig. 10) and the definitive diagnosis was a benign giant lipoma.

There was a complete clinical improvement, without any symptoms relapse or local recurrence on the remaining year of follow-up.

## 3 Discussion

Lipomas are the most common benign soft tissue neoplasm but only 1% of all lipomas present in the hand [9]. They are the most common tumour found in nerve entrapment syndromes despite nerve compression secondary to space-occupying tumours being rare [7]. However, with growth and in a low compliance soft tissue environment, such as the hand, these neoplasms can result in a fast compression of neurovascular structures, especially in the deep palmar space. Few case reports or series have reported lipomas causing secondary compression neuropathies [3,[9], [10], [11], [12], [13], [14], [15]].

Compression neuropathies in the upper extremity are common and are being recognized with increasing frequency [16]. CTS accounts for up 90% of these [17] and its highest incidence is seen in individuals aged 55–60 years [18], more commonly affecting women. Idiopathic CTS is usually bilateral. Atypical symptomatology including unilateral symptoms, sudden onset and clinical mass syndrome, as in the presented case, should raise suspicion of local causes and regular work-up should be extended. Clinical expression of secondary CTS correlates with topography of the nerve compression, but there is no evidence correlating tumour volume and symptomatology [19]. Palmar lipomas can also be associated with functional impairment in grip and digital mobility, resultant from a large size lesion or secondary to compression of the intrinsic muscles [10].

Nerve conductions studies and electromyography (EMG) are useful to determine the location and severity of the compression [16]. Standard X-rays are useful to show calcifications or bone lesions. US is a dynamic tool as it can describe the anatomy of the nerve, differentiate precisely homogeneous and hyperechoic tumours and also determine the severity of compression [20]. MRI is the preferred imaging modality when suspecting a space-occupying lesion as it demonstrates the anatomical relations and tissue characteristics and aids preoperative planning [2].

Differential diagnosis for extra-neural adipose neoplasms is mostly fibrolipoma of the median nerve and liposarcoma [3]. The high sensitivity of the MRI allows diagnostic orientations of the tumour in approximately 94% of the patients [21].

“Giant” lipoma is classically defined by a diameter greater than 5 cm and neoplasms exceeding this size should always raise suspicion for malignancy [22]. For this reason, management should be guided by the size of the lesion, leading to histopathological study as the next step. As MRI allows a confident diagnosis in case of lesions composed of only adipose tissue [11,21], excisional biopsy is the standard recommendation in the literature. It is the single treatment that allows liberation of the median nerve and effectively removes the tumour [3]. Authors suggest that a monobloc resection is the best treatment, with careful and safe dissection to reduce risk of iatrogenic lesions and disease recurrence [23]. With the histological confirmation of a benign lipoma, local recurrences are rare [19].

In the majority of the patients the pathogenesis of CTS is unclear (idiopathic) [5]. In some cases, the compression is associated with systemic factors (diabetes, pregnancy, hypothyroidism) and, generally, conventional surgical decompression of the median nerve is successful. Regarding recurrence after primary procedure, the most common factor is incomplete release of transverse carpal ligament or misdiagnosis of local underlying causes [24]. Also, endoscopic and minimal invasive techniques are being preferred for carpal tunnel release, since they demonstrate a low rate of complications and recurrence. These can lead to poorer visualization of intracanal masses [25], despite the and, when nerve compression results from local underlying causes, simple release of the median nerve may not be indicated [6].

Although rare, the surgeon must consider unusual causes of CTS when patients present with atypical symptomatology or postoperative recurrence. Further investigation may be necessary prior to intervention, usually US and MRI. In addition, these patients are bad candidates for endoscopic and minimally invasive techniques that limit surgical visualization of the nerve and possible space-occupying lesion. A correct preoperative assessment of each patient is the key for proper management, successful treatment and reduced risk of recurrence of CTS entrapment neuropathy.

## Declaration of Competing interest

The authors report no declarations of interest.

## Sources of funding

None declared.

## Ethical approval

Not applicable.

## Consent

Written informed consent was obtained from the patient for publication of this case report and accompanying images. A copy of the written consent is available for review by the Editor-in-Chief of this journal on request.

## Author contribution

Mariana Magalhães Barreira, MD MsC – Study concept, data collection, drafting, revision, approval of final manuscript.

Nuno Frederico Ramos Marques, MD MsC – Data collection, drafting, revision.

Vicente Carlos da Silva Campos, MD MsC – Revision.

Guilherme André de Paiva Marques, MD MsC – Revision.

Sérgio Rodrigues Gonçalves, MD MsC – Study concept, drafting, revision.

Sandra Vitoria Stefanova Alves, MD MsC – Study concept, revision, approval of final manuscript.

## Registration of research studies

N/A.

## Guarantor

Mariana Magalhães Barreira.

## Provenance and peer review

Not commissioned, externally peer-reviewed.
